# The Impact of Seasonality in Pasture-Based Production Systems on Milk Composition and Functionality

**DOI:** 10.3390/foods10030607

**Published:** 2021-03-12

**Authors:** Mark Timlin, John T. Tobin, André Brodkorb, Eoin G. Murphy, Pat Dillon, Deirdre Hennessy, Michael O’Donovan, Karina M. Pierce, Tom F. O’Callaghan

**Affiliations:** 1 Teagasc, Moorepark Research Centre, Fermoy, P61 C996 Co. Cork, Ireland; mark.timlin@teagasc.ie (M.T.); john.tobin@teagasc.ie (J.T.T.); andre.brodkorb@teagasc.ie (A.B.); eoin.murphy@teagasc.ie (E.G.M.); 2 School of Agriculture and Food Science, University College Dublin, Belfield, D04 V1W8 Dublin 4, Ireland; karina.pierce@ucd.ie; 3 Food for Health Ireland, Teagasc Food Research Centre, Moorepark, Fermoy, P61 C996 Co. Cork, Ireland; 4 Teagasc, Animal and Grassland Research and Innovation Centre, Fermoy, P61 P302 Co. Cork, Ireland; Pat.Dillon@teagasc.ie (P.D.); deirdre.hennessy@teagasc.ie (D.H.); Michael.ODonovan@teagasc.ie (M.O.); 5 Food for Health Ireland, University College Dublin, D04 V1W8 Dublin 4, Ireland; 6 School of Food and Nutritional Sciences, University College Cork, T12 K8AF Cork, Ireland

**Keywords:** composition, functionality, milk, seasonal, pasture, total mixed ration, dairy

## Abstract

Seasonal calving, pasture-based dairy systems are widely practiced in countries with a temperate climate and plentiful rainfall such as Ireland and New Zealand. This approach maximizes milk production from pasture and, consequently, is a low-cost, low-input dairy production system. On the other hand, the majority of global milk supply is derived from high input indoor total mixed ration systems where seasonal calving is not practiced due to the dependence on ensiled silages, grains and concentrated feeds, which are available year-round. Synchronous changes in the macro and micronutrients in milk are much more noticeable as lactation progresses through early, mid and late stages in seasonal systems compared to non-seasonal systems—which can have implications on the processability and functionality of milk.

## 1. Introduction

**Figure 1:**
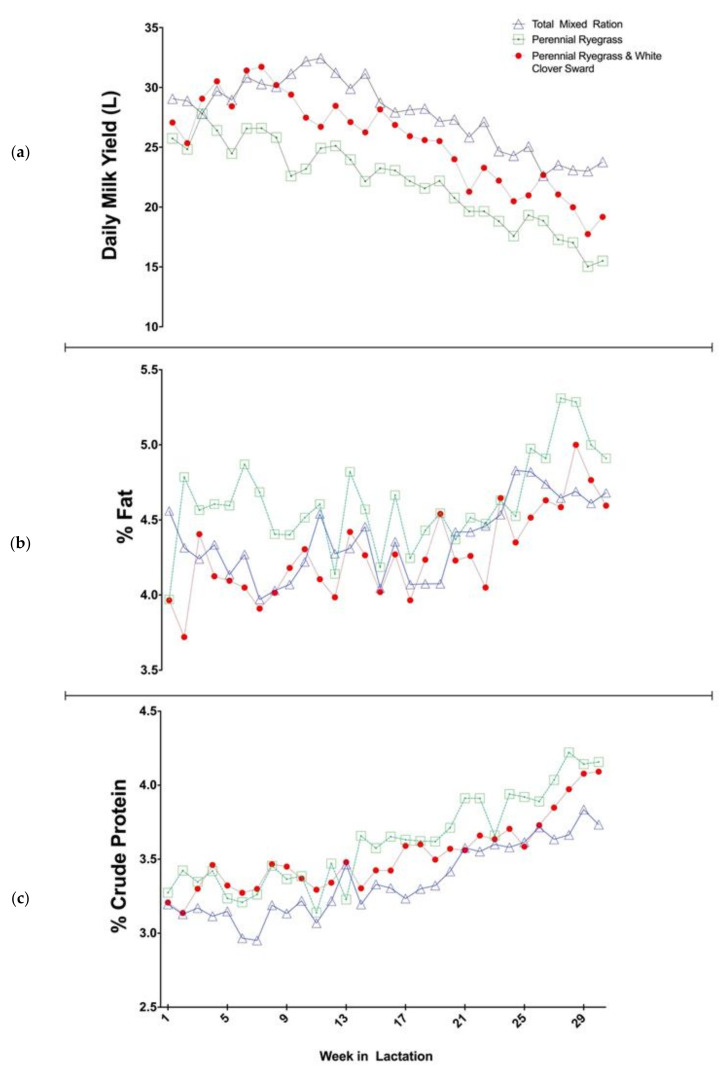
Daily milk yield (**a**), adapted from McAuliffe et al. [4], average % fat (**b**) and % crude protein (**c**), adapted with permission from O’Callaghan et al. [5], copyright 2021 Elsevier, on a weekly basis produced by seasonal calving herds consuming perennial ryegrass, perennial ryegrass with white clover, and total mixed ration (TMR) diets throughout lactation.

**Figure 2:**
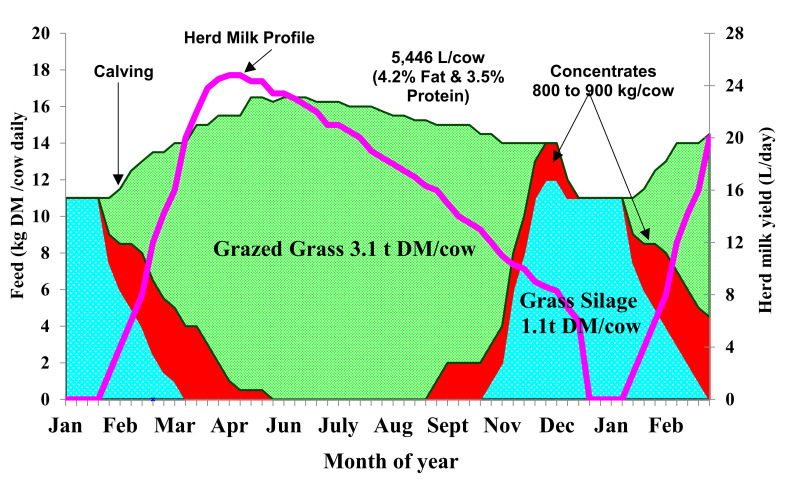
Depiction of the average Irish dairy cows’ feed budget and milk production over a 12-month period, updated from that previously reported by Dillon [21].

Regions with fertile soils, temperate climate and medium to high rainfall, such as Ireland and New Zealand, allow for seasonal calving pasture-based dairy systems, whereby cows graze outdoors for between 8 and 10 months per year [1]. In such systems, to maximize efficiency, herds calve in the spring (January–April), after which they are given access to pasture from the beginning of lactation, coinciding with the start of the grass growing season. In Ireland, 84% of dairy cows were calved in the months of January to April in 2019 [2]. Grazed grass makes up the vast majority (approximately 75%) of the diet of the lactating dairy cow in Ireland, with some concentrate supplementation and some quantities of grass silage fed in both spring and autumn. Pasture is utilized in this system due to its low cost. As the proportion of grazed grass in this system increases, the total cost of milk production is reduced [3]. Due to the compact synchronised calving period, designed to match peak lactation volumes with peak grass growth, stage of lactation (SOL) effects cause a seasonal milk supply in which the composition and quantity varies between the different stages of early, mid and late lactation. The SOL effects on the composition and quality of milk derived from each cow will occur independent of the diet consumed by the herd, as illustrated in Figure 1. Figure 1 depicts (a) the average milk yield (litres) [4], along with % fat (b) and % crude protein (c), respectively [5], from studies examining the impact of feeding system on spring calving cows milk production [4] and milk composition and quality [5]. The illustration clearly identifies and portrays a decreasing daily milk yield as well as an increase in % fat and % crude protein as the three discrete herds approach late lactation, showing independence to the diet consumed. This compositional fluctuation can result in processability issues for manufacturers, such as lower cheese yields, varying heat stability, and the inability to produce some products at all in late lactation [6]. During periods where pasture growth far exceeds that immediately required, surplus pasture is harvested and ensiled as a feed source for winter months when cows are fed indoors. As grass growth begins to slow in late autumn/early winter, cows are dried off during an 8–10 week period and receive grass based silage up until calving the following spring, when the lactation cycle begins again. Figure 2 displays the feed budget and milk production for the average Irish cow over the 12 months in this seasonal calving system. As shown in Figure 2, grazed grass is the main feed source, with the supplementation of concentrates when required during autumn and spring. Grass silage becomes the most abundant feed source during the winter months when cows are housed indoors. The effect of dietary systems has been demonstrated to have an impact on the composition and quality of milk and in particular the fatty acid (FA) profile, where substantial changes can be observed with changing nutrition [7]. The depletion of milk saturated fatty acids (SFA) and increase in the more nutritionally beneficial unsaturated fatty acids (UFA) and conjugated linoleic acids (CLA) has been shown to result from increasing levels of pasture in the diet [5,8,9,10]. This is a result of the higher concentrations of UFA in fresh grass, particularly α-linolenic acid, which is biohydrogenated into CLA (the various LA isomers which consist of a conjugated double bond arrangement [10]) in the rumen [11].

As dairy farming systems have become more intensive, the presence of pasture in the cows’ diet has declined in certain regions and a migration from pasture-based to indoor based feeding regimen has occurred at a global scale. In many countries, this indoor based feeding regimen involves a TMR feeding approach which may contain grain concentrates, forages (hay, straw, grass silage, maize silage), as well as fat, protein or salt supplements [12,13]. Factors including a lack of grazing infrastructure, land cost and availability, shorter growing seasons, access to automatic milking systems, trained labour, weather variability and a lack of grassland grazing knowledge are reasons why countries might adopt this approach [14]. Indoor TMR systems typically result in a greater management of nutrition and dry matter intake (DMI) to support the energy requirements of the high productivity dairy cows associated with TMR systems, which cannot always be achieved through pasture diets [7,15]. Over 90% of intensive dairy farms in the United States operate a TMR system [13] and it has also become more conventional in Middle Eastern countries, Japan, China, and parts of Europe despite the higher labor [7] and feed costs, the latter of which can rise to above 50% of the total operating costs [16]. Housing animals all year round offers the opportunity to condense feeding times, while the increased DMI rates allow more time for “high priority behaviour” such as resting and ruminating [15,17]. However, year round housing can result in animal welfare issues, such as increased incidences of lameness, mastitis and aggression due to space restrictions compared to grazing systems [18]. It has also been observed that TMR feeding systems result in higher quantities of enteric methane (CH_4_) emitted per kg of milk fat and protein produced [19] as well as larger overall carbon footprint per unit of milk [20]. The year-round calving schedule typically operated in this system however, results in a more consistent quantity and composition of bulk milk produced with influences such as SOL on bulk milks diminished due to calving spread. The purpose of this review is to examine available literature on the impact of pasture-based feeding strategies and seasonality on the composition and functionality of milk.

## 2. Milk Composition

Understanding the factors that affect milk composition and its subsequent processability has previously received a lot of attention. The need to maximize process efficiency and product uniformity has created a research impetus to understand changes in milk composition throughout lactation. Milk is a complex biological system, greatly influenced by factors such as SOL, feed quantity and quality, animal parity, genetics, and health [6,22,23,24]. Protein, fat, lactose, and mineral concentrations are all subject to variation linked to the aforementioned factors, altering the nutritional quality, modulating the interactions between individual constituents and their subsequent functionality.

### 2.1. Milk Fat

Fat is a very valuable component in milk due to its use in a variety of dairy products including cream, butter, full fat cheese, whole milk powder and fat filled yoghurts. Despite this wide range of ingredient uses, there has previously been a negative perception of dairy fats. This is in part due to research from Keys [25] who found a curvilinear relationship between deaths from cardiovascular disease (CVD) and the intake of dietary fats, specifically saturated fats. Despite a lack of conclusive evidence that SFA are bad for health [26,27,28,29], a modification of the internal composition of foods such as milk had been sought to include more unsaturated fats, and therefore improve the nutritional quality and health benefits available to consumers. Both feed related factors, i.e., dietary intake and seasonal or regional effects, along with animal related factors, i.e., genetics (breed and selection), SOL, mastitis and ruminal fermentation are all capable of modifying the FA composition as well as the overall quantity of milk lipids present in the milk [30].

Diet can affect total fat content at a macro level by up to 3% [31]. Studies involving TMR versus pasture diets have found that pasture derived milk had a higher fat content than that of TMR, despite TMR producing a significantly higher fat yield (linked to larger increase in milk yield) than either of the two pasture diets (ryegrass with/without white clover) [5,32]. Reynolds [33] and Drackley et al. [34] accredited the TMR diet to providing additional non-structural carbohydrates in the small intestine for digestion which is known to cause a decrease in fat concentration through an increase in overall milk yield. The incorporation of white clover into the diet causes a reduction in the fat content because of its faster rate of passage through the rumen [35], increasing the fermentation rate of the feed. This reduces the production of two substrates involved in the synthesis of milk fat by the mammary gland, rumen acetate and butyrate [36]. Another reason for lower milk fat content derived from the inclusion of white clover is the larger increase in milk yields achieved, causing a larger dilution of the fat content [5,35]. As white clover approached up to 50% of the feed composition consumed by cows, a continuous decrease in fat content could be detected, despite an increasing milk yield [35]. This results in milks derived from perennial ryegrass with white clover inclusion possessing a lower fat content than that derived from perennial ryegrass alone [5,32]. In non-seasonal herds, the highest milk fat concentration was visible in winter when supplementary silage was offered and the lowest found in summer when the herd was at pasture [6].

**Figure 3:**
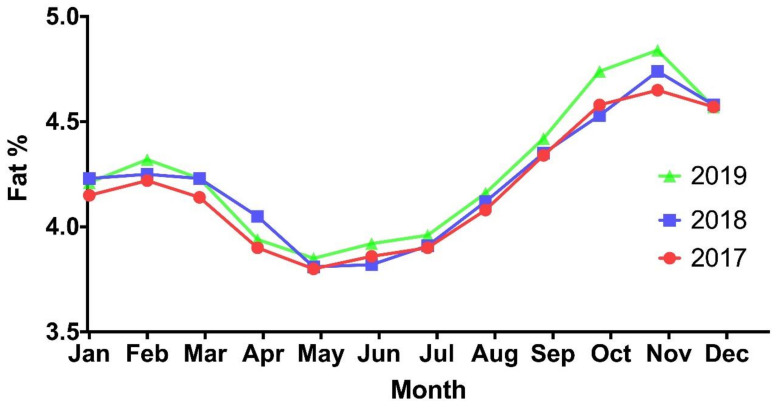
Depiction of seasonal variation in the average proportion of fat in milk produced by dairy cows in Ireland between January and December in the years 2017, 2018 and 2019, adapted from CSO data [44].

Colostrum (immediately after parturition) typically has a higher fat content than transition milks (days 1–5 postpartum) [37] and mature milks [38]. In mature milk, early lactation coincides with the lowest milk fat content, despite being the period where the highest milk fat yields are achieved [5,6,24]. After reaching peak yields in early lactation, the milk fat content will begin to increase until it reaches its peak in late lactation, however, late lactation is also where the yield of milk fat is at its lowest [5,6,24]. A study by Carty et al. [39] illustrates how fat content can increase in milk as early lactation progresses, a tendency attributed to the mobilisation of body fat reserves in the first six weeks of calving if the cow is still in a negative energy balance. The increase in fat content of seasonal herds grazing pasture in late lactation was partly attributed to the increase in fibre content of grass in addition to the physiological changes (SOL) which cause a concentrating effect due to a decreasing total milk yield [40]. The occurrence of milk fat depression (MFD) in Irish herds partaking in milk recording was correlated with the month of May, when cows consumed an abundance of fresh pasture rich in polyunsaturated fatty acids (PUFA) [39]. Partial biohydrogenation of these dietary PUFA’s results in the production of over twenty CLA isomers, three of which are known to inhibit milk fat expression (*trans*-10, *cis*-12 CLA; *trans*-9, *cis*-11; and *cis*-10, *trans*-12) [41,42]. These isomers are transported to the mammary glands via the animals blood where they impair the production of essential fat synthesis enzymes, inhibiting milk fat synthesis [41]. This was quite often noticeable in May because of the low fibre, and high PUFA content of relatively young fresh pasture, prior to grass maturing and going to seed later in the summer [39]. Cows were also less likely to undergo MFD as they transitioned indoors and began to incorporate grass silage into their diet [43]. This was accredited to the storage of pasture as grass silage causing a reduction in the PUFA levels of the feed and therefore reducing the animal’s intake of PUFA. Figure 3 [44] illustrates the variation in milk fat content from Ireland’s bulk milk supply during 2017, 2018 and 2019. Lowest fat contents can be seen when the majority of cows are approaching peak lactation during April/May [5,6,24] and consuming pasture with large amounts of PUFA [39]. From May onwards, there is an increase in the milk fat content instigated by a higher fiber content in the pasture as well as decreasing milk yields caused by the advancing SOL [40].

The effect of cow breed on fat production is evident in a study by White et al. [10]. This research indicates that Jersey cows consuming both TMR and pasture diets produced milk with a significantly higher milk fat content than Holsteins on the same diets. Carroll et al. [45] and Drackley et al. [34] also noticed a larger milk fat yield in Holsteins than both Jerseys and Brown Swiss cows but, because of the larger milk yield, this subsequently resulted in a lower fat content in Holsteins.

#### 2.1.1. Milk Fat Globule Membrane

Triacylglycerol constitutes over 98% of the fat droplet along with diacylglycerol and monoacylglycerol [46]. The remainder of the fat globule is mostly comprised of cholesterol, proteins and glycoproteins which are embedded in a phospholipid layer called a milk fat globule membrane (MFGM), giving the fat droplet a bipolar structure [47]. This provides the globules with greater stability and emulsion properties while suspended in the milk serum phase [48]. Milk possessing smaller milk fat globules (MFG) provides a larger proportion of MFGM due to their superior surface area to volume ratio exhibited, providing a more stable suspension in the milk serum compared to that with a larger MFG [49].

The MFGM plays an important role in protecting the core milk fat from developing light activated off flavours, hydrolytic rancidity and “oiling off”, characterized by the presence of fat globules on the surface of tea or coffee [22]. Damage to the membrane through the collection and processing of milk may increase the likelihood of each occurring. If damage does occur to the MFGM, caseins and whey adsorbed to the surface of fat globules creates an artificial membrane. This new artificial membrane does little to prevent the subsequent hydrolytic rancidity and is therefore particularly evident in homogenized milk [50]. The breakdown of the fat globules protective layer also causes the fat to be more susceptible to sunlight oxidized flavour [51]. The integrity of the MFGM dictates the participation of the MFG in gelation [52]. Michalski et al. [50] reported no interaction between undamaged MFGM and the casein network during gelation but reactions could be observed when the fat globules underwent mechanical treatment and the structural integrity of the membrane was reduced. Dewettinck et al. [51] outlines the desirable functions of MFGM in applications such as baking, chocolate production and drug delivery due to its emulsifying and water retention properties. Isolation of MFGM from buttermilk and butter serum has therefore become increasingly valuable to milk processors owing to the high quantities present. The water retention properties have also been used to produce cheese with a higher moisture content by using smaller MFG which provides a larger MFGM/milk fat ratio [53].

The majority of studies examining the composition of milk fat have focused on the overall FA profile of the milk, with very limited data on the variation in relative abundance of MFGM due to diet or SOL. Much of the existing research focusing on MFGM has linked its relative abundance to the size of the MFG. In a study related to the dietary changes to milk lipids, Lopez et al. [54] discovered that a PUFA rich diet produced fat globules with a larger proportion of phospholipids compared to milk fat derived from a diet with a higher SFA content. This was accredited to the smaller fat globules produced by the PUFA rich diet, resulting in fat globules with a larger surface area/volume ratio, where phospholipids are more abundant due to their presence in the MFGM layer at the surface of the fat globule. The phospholipids derived from the PUFA diet in this study consisted of a larger proportion of long chain and very long chain SFA, reduced C16:0 and increased fractions of C18:0 [54]. Research from Walker et al. [55] recognized a higher phospholipid content in milk derived during late lactation than during any other stage, and again, this was linked to a reduced MFG size. Liu et al. [56] also documented peak phospholipid contents in late lactation despite variation between the individual animals sampled. Bitman et al. [57] documented a decline in phospholipid and cholesterol concentrations as lactation progressed, however, samples for this study were only collected until the 180th day postpartum (mid lactation).

#### 2.1.2. Fatty Acid Profile

Over 400 FA have been identified in milk; some of these are synthesized in the mammary gland (de novo synthesis) to produce C4:0–C14:0 along with some C16:0 [58]. The remaining FA, which typically are of C16 chain length and longer, originate from both the dietary intake of FA and the mobilization of adipose tissues triacylglycerols [59]. These FA pass into the bloodstream from the digestive tract, where they can be taken up by the mammary cells which incorporates them into the milk fat [58,59]. Almost all of these FA combine to form different structures within the fat globule, the majority being triacylglycerols, with each structure varying in FA combinations, molecular weight and saturation [46]. Triacylglycerols are formed when FA bind to sn-Glycerol-3-phosphate in three potential locations: sn-1, sn-2 and sn-3. Enzymic mechanisms control the FA esterification at each sn-position [46,60]. Long chain FA (C16 upwards) are esterified at the sn-1, medium chain FA (C8–C14) along with some C16 are esterified at the sn-2 position and short chain FA (C4–C8) along with some C16 and C18:1 are esterified at sn-3 [61]. The FA profile of milk is an important factor affecting the nutritional and functional properties of milk.

Epidemiological studies have generated strong positive correlations between incidences of cardiovascular disease and consumption of SFA in the past [62]. This led to a reduction in the consumption of products containing dietary SFA, including dairy products, over the last 30 years due to reports of their high SFA levels [63]. More recent studies however, have shown not all SFA are equal in this regard and each structure presents a different biological function in the body [64]. A study from Legrand et al. [65] has suggested that only palmitic acid (C16:0) causes a concern with respect to cardiovascular disease and cholesterol risk but only when consumed at 8–10% of daily energy [26,27,28,29].

Milk fat contains relatively high quantities of UFA which play a role in maintaining normal physiological conditions in human health [66]. They are also beneficial to dairy processors because of the association between the concentration of PUFA and butter spreadability, resulting from lower melting points of PUFA [67]. The second most abundant FA in milk is an UFA in the form of oleic acid (C18:1) which accounts for up to 25% of total fat [64]. C18:1 is formed from both biohydrogenation of PUFA, particularly linolenic and linoleic acid (18:2*n*-6; LA), as well as ∆^9^-desaturation of stearic acid (C18:0) in the udder due to the presence of the stearate desaturate enzyme present in the mammary tissues [59,68]. The softness and plasticity of C18:1 gives it an important role in improving the spreadability of butter [31]. C18:1 has also been linked to improve cancer drug effectiveness along with inhibiting oncogenes role in causing breast cancer in humans [69]. Other examples of UFA are omega 6 (*n*-6) and omega 3 (*n*-3). Their precursors, LA and alpha-linolenic acid (C18:3*n*-3; ALA), are considered essential FA as they are not synthesized in the body [66,70]. Research has demonstrated a beneficial effect of consuming LA and ALA in preventing heart disease and other cardiovascular events [70,71]. Large quantities of omega 6 FA have contributed to excess chronic inflammation [72]. This has encouraged research into the ‘ideal’ ratio of *n*-6/*n*-3 FA in the diet which Haug et al. [73] has suggested to be 1–2:1. Despite this, the ratio of *n*-6/*n*-3 has increased to as high as 14:1 in Western diets in recent years, and has been linked to increased health risks including cancer and obesity [66,74]. Optimizing the production and consumption of both *n*-6 and *n*-3 would be highly beneficial to promoting good health status.

Lipoprotein lipase presence in milk causes the lipolysis of triglycerides, resulting in the formation of free fatty acids (FFA) and partial glycerides [75]. Lipoprotein lipase is involved in the synthesis of milkfat triglycerides in the mammary gland and its presence in milk is regarded as “spillover” as it has no known biological purpose [75]. Lipolysis may naturally occur in raw milk due to poor quality feed, mastitis, late lactation or through mechanical mechanisms during processing such as the physical breaking of the membrane of the globule with agitation, homogenization or pumping [75]. The likelihood of lipolysis occurring before consumption of the dairy product can cause concerns for dairy manufacturers as it can result in unpleasant flavours in the product [76]. As FFA levels begin to approach 1.5 mmol/L, the milks steam foaming ability begins to deteriorate, a decrease in the surface tension occurs, and (due to the relatively low flavour threshold of short and medium chain FFA) rancid off flavours of milk become present [75,77,78].

Using nutrition to control the FA profile of milk fat has received extensive attention over the last three decades. Reducing the quantity of SFA, improving the properties of manufactured dairy products and enhancing the concentrations of “good” FA have all received particular attention in this regard. Particular variation has been shown in C18:1 as a function of diet. The proportion of C18:1 increased from 14–18% up to 22–24% while transitioning from indoor winter feed to outdoor pasture [68], while the proportion of C16:0 reduced with increasing proportions of fresh grass [9]. Studies from O’Callaghan et al. [5] and O’Callaghan et al. [79] similarly identified TMR derived milk and butter possessed significantly higher C16:0 content than that from pasture diets, resulting in a larger C16:0/C18:1 ratio. The correlation between C16:0/C18:1 ratio and texture, which has been suggested to increase hardness and reduce spreadability of butter as the C16:0/C18:1 ratio increases [9], as was demonstrated in O’Callaghan et al. [79] study. Pasture derived butters, whose C16:0/C18:1 ratio was lower than TMR, scored significantly higher for its texture attributes than TMR derived butters using sensory ranking descriptive analysis scores [79]. Desaturation of C18:0 into C18:1 has been shown to be inhibited by an increased intake of PUFA or *trans*-FA, while also reducing fat yield [80]. The large intakes of PUFA associated with pasture grazing have also been demonstrated to produce milk fat with a large proportion of ALA and rumenic acid (RA; C18:2 *cis*-9, *trans*-11 CLA) [81]. Human consumption studies have reported the numerous health benefits associated with the consumption of RA including the reduction of plasma triacylglycerol, LDL-cholesterol/HDL-cholesterol and total cholesterol/HDL-cholesterol in healthy men [82]. Improvements to cardiovascular health, anti-carcinogenic properties and improved immune function have also been associated with RA consumption [83]. The larger proportions of PUFA from pasture-based diets results in the synthesis of up to twice as much RA per 100 g of milk fat through ruminal biohydrogenation than that of TMR based diets [5,79,84]. The ruminal biohydrogenation of these PUFA supply’s precursor, vaccenic acid (C18:1) and small quantities of RA to the mammary glands [84]. The mammary tissue then forms the majority of the RA in milk fat through the ∆^9^-desaturase of C18:1 [85]. Zero grazing reportedly reduces the proportions of RA in milk in comparison to grazing animals [86], while both hay and maize silage produce milk with a lower RA content than that of fresh pasture, but a higher RA content than grass silage derived milks [68].

A study conducted in Switzerland identified higher concentrations of milk CLA, branched chain FA and *n*-3 FA as the proportion of herbage consumed increased [87]. Studies from both Ireland [5] and Portugal have demonstrated pasture-based diets positive influence on reducing milks *n*-6/*n*-3 ratio, producing milk with a higher *n-3* content and lower *n*-6 content than cows fed a TMR diet. Benbrook et al. [88] similarly reported a lower ratio when cows are fed an organic pasture diet compared to a conventional diet in the United States while Chilliard et al. [89] associated an increase in the *n*-6/*n*-3 ratio of milk with the increasing quantity of maize silage used as a replacement to grass silage. This is a result of the much higher abundance of ALA, an *n-3* FA, in fresh pasture than in maize based or high concentrate diets [68]. As a long chain FA, the larger proportions of ALA entering the digestive tract from pasture, allows larger proportions to pass into the blood stream and become incorporated into the milk fat by the mammary glands [58]. Pasture derived milks have also demonstrated increased concentrations of CLA when compared to milks derived from winter diets [90,91,92]. Milk CLA concentrations were higher in grass silage produced from fresh leafy grass than from mature seedy grass [93]. This was linked to the high quality of dietary PUFA in fresh leafy grass which is generally in abundance in May before going into a reproductive stem in June/July [93]. Reductions in the CLA content, as well as proportions of C16:0, C18:0, C18:1, and C18:3, in pasture have been reported from May as the grass becomes more mature, until July and August, when the proportion of grass leaf begins to increase again [94,95].

Cows consuming low fibre diets associated with fresh green leafy grass produced larger quantities of short and medium chain FA than high fibre diets but produced lower concentrations of long chain and *trans* FA [31,96]. The addition of an emulsified CLA supplement (94.5% *trans*-10,*cis*-12 CLA), in low fibre diets led to an increased concentration of *trans*-10 isomers in milk [80]. The presence of these *trans*-10 isomers in milk, particularly *trans*-10 C18:1, have been linked as a marker to a reduction in milk fat synthesis as well as the occurrence of MFD [41,97,98,99]. It has been suggested that this fat reduction is a consequence of a reduced de novo synthesis of small and medium chain FA within the mammary cells [100]. Low fibre diets have also been shown to induce subclinical ruminal acidosis (SARA) in cows, causing this reduction in de novo synthesized FA, as well as the overall milk fat content [101,102]. This is as a result of lactic acid and organic FA, such as volatile fatty acids (VFA), accumulating in the rumen, causing a reduction of the rumen pH at faster rate than rumen buffering can counteract [103]. Dietary sources with high rumen digestibility, such as grains and fresh pastures, increase the production of VFA in the rumen, increasing the likelihood of SARA occurring [103,104].

Kelsey et al. [105] demonstrated that breed, parity and SOL have very little influence on CLA variables including the proportion of CLA in milk fat and the CLA desaturase index. In New Zealand, Auldist et al. [6] determined that changes in the FA profile was a reflection of the energy balance of the cow rather than SOL. This was characterised by a reduced synthesis of small and medium chain FA when the cows were incapable of consuming the necessary dry matter to match their energy requirements in early lactation [6,106]. To compensate for this, increased quantities of FA were mobilized from adipose tissue, augmenting the number of long chain FA present [6,106]. This could be observed in a study from Canada where proportions of C6:0, C8:0, C10:0, C11:0, C12:0, C14:0 and C15:0 were lowest in early lactation, but increased until day 100 where they remained constant thereafter [107]. The proportion of C16:1, C17:0, C18:0 and C18:1 in milk fat was highest at the beginning of lactation but decreased throughout early lactation until day 100 [107]. Similarly, Gross et al. [108] observed lowest proportions of small and medium chain FA at the beginning of early lactation up until week 12 of lactation, with proportions of C18:1 decreasing over the same time period. The spring flush on a seasonal farm in Poland, resulted in an increase in long chain FA as cows transitioned onto fresh herbage [109]. In Ireland, early lactation milk was characterised by long chain FA C18:1 and C18:0 independent of diet [5]. Mid and late lactation milks however, were characterised by eicosatrienoic acid (C20:3), erucic acid (C22:1), palmitic acid (C16:0) and LA (C18:2) when consuming TMR and pentadecanoic acid (C15:0), tridecanoic acid (C13:0), tricosanoic acid (C23:0), linolenic acid (C18:3), CLA (C18:2 *cis*-9,*trans*-11), arachidonic acid (C20:4), undecanoic acid C11:0, and linolelaidic acid (C18:2) when consuming pasture diets [5]. O’Callaghan et al. [37] demonstrated an increasing UFA content and decreasing SFA content between colostrum and milk 5 days post parturition.

Cow breed has also been demonstrated to impact milk fat composition. Milk produced from Jerseys has been demonstrated to have increased proportions of short chain FA than that produced from Holsteins and Brown Swiss, specifically C4:0, C5:0, C6:0 and C7:0 which have been linked to larger MFG [45]. An increase in C12:0, C14:0 and C16:0 esterified in the sn-2 position is also higher among Jerseys, suggesting there is an increased level of de novo synthesis of FA in Jerseys compared to Brown Swiss and Holsteins [45,110]. In Soyeurt et al. [111] study, the FA profile of Jersey and Holstein cows varied significantly, with C16:1 *cis*-9 content being the only non-significant difference between the two breeds. One significant difference observed between the two FA profiles was the Jerseys ability to produce milk with a SFA profile of higher nutritional quality than that of Holsteins [111]. Isomers of CLA (C18:2 *cis*-9, *trans*-11 and C18:2 *trans*-10, *cis*-12), which have been linked with the ability to help prevent cancer [112], have been observed in higher concentrations in milk from Holsteins than that of Jerseys [10]. A 13–15% higher C18:1 was also observed in milk produced by Holstein compared to Jersey breeds [69,113].

### 2.2. Protein

The protein content of bovine milk can vary from 2.8% to 4.6% depending on four main factors including (1) management, (2) health, (3) nutrition and (4) genetics [114,115]. Bovine milk protein’s natural function is to supply essential amino acids to young mammals through its two primary protein groups; casein (~80%) and whey (~20%), which aid in the development of muscular tissues [22]. Milk proteins are considered a very high quality protein source due to their high concentration of branched chain amino acids (valine, leucine and isoleucine) which play important roles in metabolic functions, such as muscle synthesis [116].

The casein in bovine milk primarily forms the building blocks of large colloidal particles called casein micelles [117]. The other constituents of these micelles are colloidal calcium phosphate (CCP) which occupies ~6% of its structure [22]. Casein’s primary function in milk is to deliver insoluble calcium phosphate to neonates [118]. To do this, casein micelles are required to be a complex mix of hydrophobic and hydrophilic elements which allow the delivery of insoluble substances while remaining suspended in the milk [118]. The micelles ability to coagulate in the stomach of the new-born through specific enzymatic cleavage allows the essential milk components to be digested [119]. In bovine milk, caseins are made up of four main subgroups; α_s1_-casein, α_s2_-casein, β-casein and κ-casein [22]. The κ-casein subgroup represents approximately 13% of total casein and is located in areas on the outside of the micelle where it stabilizes calcium-sensitive casein subgroups (α_s1_-casein, α_s2_-casein, β-casein) that make up the remaining 87% of the micelle [22,119]. This calcium sensitive core is responsible for entrapping the majority of water in the micelle through interior channels, while the κ-casein ‘brush’ on the surface of the micelle accounts for approximately 30% of the water associated with the micelle [120,121]. κ-Casein plays a primary role in the coagulation properties of milk, greatly improving the coagulation properties, due to its ability to reduce steric repulsion and net negative charge between micelles in response to pH change to the isoelectric point of caseins [122] and when enzymatically hydrolysed [123,124]. The degrees to which the coagulation properties are improved are dependent on the κ-casein allele. The larger hydrophilic surface area of κ-casein B allele reduces the critical level of κ-casein hydrolysis of the micelle required to induce coagulation, improving the micelles gelation properties in comparison to the A allele commonly exhibited in Holstein cows [125,126,127]. The proportion of β-casein present in a micelle has been shown to be proportional to the micelle size suggesting it is mainly located beneath the surface of the micelle [121]. This is implied by a larger volume to surface area ratio which occurs with larger micelles, allowing a higher proportion of constituents on the interior in comparison to the exterior.

The other main constituent of milk protein is whey protein (also known as serum protein or non-casein nitrogen (NCN)) comprising ~20% of milk protein [128]. In contrast to casein, whey remains soluble, allowing a rapid delivery of amino acids to the gut [115]. Whey proteins typically show a relatively large susceptibility to denaturation due to their high levels of secondary and tertiary structures [22]. These denatured whey proteins lead to the formation of sulphur compounds which give milk its “cooked” and “cabbagy” off flavours after heating [22]. β-Lactoglobulin (β-lg) is the main constituent of bovine whey protein with approximately 50% of whey taking this form [22,129,130]. Due to its high cysteine content, β-lg is important in milk manufacturing, which, upon heat denaturation, causes a disulphide-linked complex with κ-casein improving rennet coagulation and affecting heat stability [22]. It is also seen as a cost effective substitute for egg whites, whose foaming properties depend on ovalbumin and globulins [131], due to its excellent foaming ability [132]. The high solubility of β-lg has also resulted in its use as an ingredient in protein fortified beverages [132]. α-Lactalbumin (α-la) is the second most abundant whey protein in bovine milk comprising 4% of the total protein and 20% of whey protein [22]. α-Lactalbumin is also a great source of two essential amino acids, tryptophan and cysteine, which are precursors of serotonin and glutathione [132]. α-Lactalbumin has the ability to promote apoptosis of tumour cells while also showing anti-proliferative effects on colon adenocarcinoma [133]. Its structure and folding is highly dependent on calcium, particularly at a low pH [132]. In the absence of calcium, the structure of (α-la) can be altered at temperatures as low as 43 °C, 25 °C lower than the 68 °C required to alter the structure when calcium is present [132]. Serum albumin, lactoferrin, immunoglobins, lysozymes, enzymes and hormones compose the remaining constituents of bovine whey proteins [134].

Post translational modifications of proteins occur in the endoplasmic reticulum and the Golgi apparatus of the mammary gland [135]. Post translational modifications are responsible for the addition of covalently bound prosthetic groups onto amino acids. Examples include glycosylation and phosphorylation. Prosthetic groups are often charged at neutral pH, hence affecting the overall charge of the proteins. Glycosylation is essential for correct protein folding (3D) and conformation, solubility, intracellular transport or tissue targeting [136]. Glycosylation of proteins may also affect the proteolysis during gastrointestinal digestion [135], while their tendency to bind iron [137,138], saccharides [139] and calcium [135] is essential in assisting to build the immune and digestive systems of the newborn [140]. Phosphorylation is essential for binding both organic and inorganic calcium to the protein, allowing the delivery of large amounts of minerals to the newborn, through the casein micelle [139]. This ability to bind calcium does however impact the rennet coagulation properties of the milk. The presence of more phosphorylated α_S1_-casein and α_S2_-casein variants results in an increased risk of non-coagulating milk occurrence [141].

Milk urea nitrogen (MUN) contributes to approximately half of the total non-protein nitrogen (NPN) in milk [142], with the remaining half consisting of small peptides and free amino acids [143]. Due to its rapid equilibration with blood urea nitrogen, MUN is considered a good indicator of crude protein conversion in cows [144]. Urea is mainly produced because of the inability of cows to build protein reserves, rendering them unable to adjust their protein supply depending on their protein requirements and current intake [145]. Milk urea is inversely correlated with nitrogen conversion efficiency and is formed from gluconeogenesis of amino acids, surplus quantities of rumen degraded proteins and excessive amounts of true protein digested in the small intestine [146]. This creates excess ammonia which is then synthesized in the liver [147]. A large energy intake is required to synthesize the excess ammonia into urea, which is then excreted in high proportions as waste through the urine of the cow (urinary urea nitrogen; UUN), and through the mammary glands (MUN), where it is encompassed into the milk. The result of this is a negative correlation relative to the energy available for milk production in the ruminant [148]. Increased proportions of urea in milk can also increase heat coagulation times (HCT) [149], making milk more suitable for high heat treatments like ultra-high temperature (UHT), but concurrently reducing the nutritional quality of the milk protein.

Due to its dependency on crude protein intake, MUN content is primarily affected by the animal’s diet. Keim et al. [148] on review of the topic reported the nitrogen conversion efficiency to be as low as 13–31% in milk produced from cows consuming fresh pasture with high crude protein contents, however, this can increase to between 40–45% when cows consume a diet containing a lower crude protein content. The research of O’Callaghan et al. [150] on rumen metabolism highlights the different efficiencies of nitrogen conversion in the rumen as cows consume various diets, which directly affects the protein quality synthesized in the mammary epithelial cells. Good nitrogen conversion efficiency should provide low levels of MUN, and favour the production of high quality proteins such as whey and casein. In O’Callaghan et al. [150], TMR and perennial ryegrass with white clover inclusion produced increased concentrations of MUN, suggesting low nitrogen metabolism efficiency (NME) within these diets. Low NME has been outlined to increase waste on farms through a larger expulsion of nitrogen as urea during urination [147,151,152]. Additionally, poorer NME causes issues for processors regarding a lower product yield resulting from a larger contribution of NPN to the overall nitrogen content as well as the lower true protein content. O’Callaghan et al. [5] demonstrated that milk derived from a perennial ryegrass with white clover inclusion diet possessed NPN concentrations (for which urea accounts for up to 48% [153]) higher than both TMR and perennial ryegrass alone whose NPN concentrations were the same. Similarly, Harris et al. [154] showed an increased white clover content in the diet was linked to larger proportions of urea, NPN and NCN in the derived milks which caused an inverse effect on the casein to total nitrogen ratio. The authors accredited this to high levels of dietary crude protein present in white clover herbage which was not fully utilized and therefore wasted. Pasture quality also affects MUN, with lowest MUN concentrations identified in summer, correlating with a depressed protein intake from pasture during this period [6].

Studies by Couvreur et al. [9], and O’Callaghan et al. [5] have identified higher crude protein and true protein (TP) contents in milk produced from pasture-based diets compared to an indoor TMR diet. The TMR diet did however record higher yields for total protein as a result of increased milk yields. The larger increase in milk yield compared to protein yield results in a ‘dilution’ effect, reducing the concentration of milk protein [31]. This effect resulted in improved cheese yields in milk derived from pasture due to the higher proportion of TP per litre of milk [59]. The inclusion of silage in a pasture diet also reduces the protein concentration as evident when cows are housed indoors due to poor grass growth and grazing conditions at the beginning and end of the milking season [40]. Auldist et al. [155] observed higher concentrations of whey proteins in milk derived from pasture than TMR. Kefford et al. [23] demonstrated an increase in whey protein concentration along with a concurrent reduction in casein number ((casein/total protein) × 100) occurred due to lower energy diets. O’Callaghan et al. [5] reported that TMR diets resulted in the production of milk with lower casein content than that of pasture-based diets (perennial ryegrass/perennial ryegrass with white clover). In similar studies, both casein concentrations [32] and casein/protein ratios [155] were both lower in milk derived from TMR diets than the perennial ryegrass alternative. In countries where fresh leafy grass is not as abundant such as Argentina, cows consuming TMR produced milk with a larger protein content compared to cows grazing outdoors on a pasture-based diet of winter oats [156].

**Figure 4:**
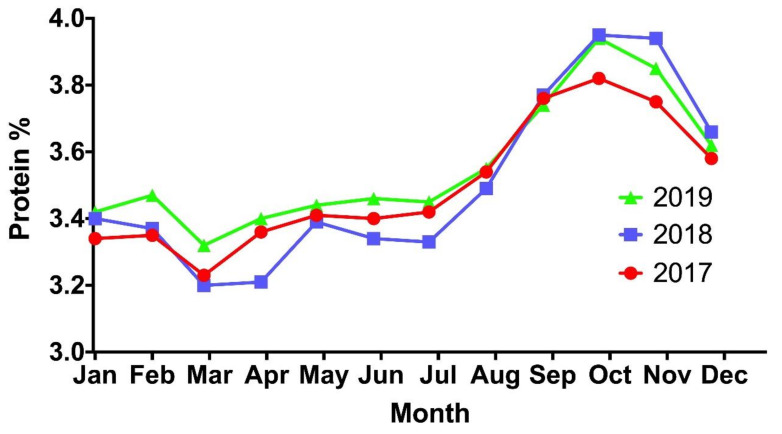
Depiction of the average protein concentration in milk produced by Irish cows between January and December in the years 2017, 2018 and 2019, adapted from CSO data [44].

Seasonal change to protein composition is primarily attributed to SOL effects. One noticeable consequence of this is the reduction in milk protein content postpartum until peak yield in early lactation [5,6,24]. As milk yield declines and cows progress into late lactation, an increase in the protein content of milk can be observed [5,6,24,40]. The extent of this effect in countries whose herds implement a synchronized SOL, such as Ireland, can be seen in Figure 4 [44]. As is visible in the illustration, the protein content of the milk is low during the early lactation period (March/April) before reaching its highest proportion in the late lactation period (October/November). An increase in whey protein content in late lactation has been linked to the loosening of mammary epithelial cells [6], allowing a greater permeability through which whey proteins can pass, thus increasing their concentration in milk, similar to the effect caused by both mastitis and high plasmin activity [157]. This trend has been investigated by Auldist et al. [155] who suggested whey content should only increase when the cows daily milk yield decreases below 5 kg/cow daily. A previous study by Auldist et al. [6] did identify higher concentrations of whey during late lactation and lower concentrations during early lactation. On examination of Irish spring calving systems, O’Callaghan et al. [5] similarly identified highest concentrations of both whey and casein in October, coinciding with the latter stages of the Irish seasonally calved herds lactation, and lowest concentrations in March/April, coinciding with the peak milk yield of the herd in early lactation [5]. This trend was identified across each of the three diets (perennial ryegrass, perennial ryegrass with white clover and TMR) studied for the spring calving herds, providing evidence of seasonal variation in whey and casein protein concentrations due to SOL [5]. A reduction in the availability of amino acids from pasture during autumn and spring limited the availability and production of casein in the derived milks during these periods [6]. The relative proportions of casein fractions α_s_-casein and β-casein follow different tendencies during the first two months of lactation. Kroeker et al. [158] highlighted the rapid reduction in the relative percentage of α-casein while a concurrent, reciprocal rise in the relative proportion of β-casein, both of which stabilized approximately two months postpartum. α_s1_-Casein was most abundant in early lactation according Li et al. [24] while Bernabucci et al. [159] similarly noticed the ratio of α-casein to β-casein decreased from spring-summer through until autumn-winter. α-Lactalbumin was likewise shown to decrease from early season right through until late season in seasonally calved herds in New Zealand [24] and Denmark [160]. Ostersen et al. [160] also observed β-lactoglobulin generally followed the same parabolic trend as the total protein content. Colostrum has been shown to be rich in glycosylated proteins [136], particularly lactoferrin [161] and immunoglobulin [136,162,163] with sharp declines observed by day 5 postpartum [161]. Lactoferrin glycosylation could be characterised into 3 distinct phases in O’Riordan et al. [161] study; colostrum (days 1–4 postpartum), transition milk (days 5–30 postpartum) and mature milk (days 31–90 postpartum). The proportion of glycosylated κ-casein (G-κCN) was observed to increase in Danish Holsteins with days in lactation (DIL) [164], however, a decrease in G-κCN was also observed in Danish Jerseys. The same study identified a decrease in the degree of phosphorylation of α_S1_-casein and α_S2_-casein between 189 and 218 DIL before increasing again in milk produced after 218 DIL [164].

### 2.3. Lactose

Lactose is the main carbohydrate component in milk and may exist in two anomeric forms, α-lactose and β-lactose [22]. Lactose consists of a β1-4 glycosidic bond linking a d-galactose and d-glucose together and is therefore a disaccharide [165]. As well as providing approximately 30% of the caloric value in bovine milk, lactose plays a role in controlling the osmotic pressure of milk along with other low molecular weight constituents including milk salts [165]. In milk manufacturing, lactose is required for fermentation of lactic acid bacteria when producing yogurt and cheese [49].

During the synthesis of lactose in the mammary epithelial cells via the glucose molecules absorbed from blood, water is drawn into the Golgi vesicles, causing a dilution of the majority of constituents in milk [165,166]. This results in a direct relationship between lactose and milk yield (and an inverse relationship to proteins, lipids and minerals), which increases until peak milk yield is reached, approximately 6–8 weeks after calving, where it then declines until the cow is dried off [22]. Auldist et al. [6], O’Callaghan et al. [5] and O’Neill et al. [19] all documented the reduction in lactose content as the cow approaches late lactation which Phelan et al. [167] attributes to a compensating effect of lactose due to an increase in milk salts passively diffused through the mammary epithelial cells. A similar occurrence happens when the mammary epithelial cells become damaged and leak due to inflammation caused by mastitis, again increasing the minerals diffusing into milk which causes a reduction in lactose, ensuring a stable osmotic pressure between the milk and blood [168,169]. Sneddon et al. [170] identified no significant difference in lactose content from milk produced between differing breeds.

Pasture derived milk has demonstrated lower lactose contents than milks from non-pasture diets in both Switzerland [87] and Ireland [19]. The increase in lactose produced from TMR diets is associated with the higher energy supplied from starch [171,172]. These results contrasted with O’Callaghan et al. [5], who found no significant difference in lactose levels between pasture and TMR feeding systems for Irish spring calved cows. A study by Auldist et al. [6] which involved distributing calving evenly throughout the year, reported that lactose had a larger composition in milk produced in summer than that when feed was subsidised with ensiled grass silage during winter.

### 2.4. Vitamins and Minerals

Despite generally being consumed in trace amounts, both vitamins and minerals are essential components of foods and a healthy diet. The body’s inability to synthesize vitamins makes their consumption essential for growth and maintenance of health [22]. Vitamins have roles in improving the immune defence system (vitamin C and E), maintaining healthy skin (vitamin A) and decreasing the risk of cancer and coronary heart disease (vitamin E) [73,173]. In comparison to other foods, milk provides a cheap and convenient source of vitamins with up to 100% of the recommended daily intakes of riboflavin (vitamin B_2_) and cobalamin (vitamin B_12)_ and 60% of vitamin A met by 5–7 year olds drinking 1 pint of milk [174].

Minerals assist in the development and function of cellular systems such as the immune system as well as playing a role in part of every cell, fluid, tissue and organ [175,176]. Their function is not just limited to providing nourishment for growth, as they also play a structural role in milk. Both calcium and phosphorus provide structure and stability to casein micelles as CCP bound to casein submicelles [177,178]. This has resulted in a positive correlation between calcium and phosphorus with casein [177]. When CCP’s structure is altered, the stability of the micelle may be effected and this can result in the aggregation of the micelles or dissociation of its components [179]. One mineral capable of disrupting CCP’s structure, and therefore instigating micellar change, is citrate [180]. Its strong affinity for calcium, which it bonds with at a ratio of 1:1, causes a decrease in CCP along with a proportionate increase in serum calcium [22].

Diet has been correlated with slight variations of the mineral content in bovine milk. This relatively minimal effect noticed has been attributed to the ‘reservoir’ of minerals associated with the animals skeleton [22]. The maternal skeleton is known to demineralize during periods where intestinal mineral absorption fails to meet the neonates mineral requirements, compensating the deficit [181]. The skeletal demineralisation most commonly occurs during times of large minerals demand during lactation, particularly in early lactation and colostrum production [182]. The mineral reserves are then replenished when mineral demand falls below that absorbed in the intestine [182]. Iodine, unlike most other minerals in milk, is highly dependent on diet, with conversion of iodine from feed to milk as high as 27% depending on the iodine intake of the animal [183]. Indoor feeding diets during winter have produced milk with higher iodine concentrations than outdoor summer diets [184]. Milk resulting from a diet consisting of perennial ryegrass provided higher concentrations of calcium when compared to an indoor TMR and perennial ryegrass with white clover in spring calved herds [32]. In the same study, milk derived from perennial ryegrass also provided ~3–5 mg/100 g more phosphorus than milk produced from diets consisting of TMR or perennial ryegrass with white clover, while magnesium and sodium underwent very little change due to feeding system. Deficiencies in citrate content have been identified in milk derived from high concentrate diets which lack in roughage (fibrous indigestible material in foodstuffs) [185]. An increased amount of de novo FA synthesis caused by high concentrate diets was acknowledged as the cause of this citrate deficiency as citrate plays a role in forming a reducing equivalent, NADPH (Nicotinamide adenine dinucleotide phosphate) [186]. Through the use of supplementary fats, Banks et al. [187] decreased the de novo synthesis required for digestion and reported a proportional increase in citrate contents of the resulting milk. Citrates dependency on diet is also evident in studies by Keogh et al. [185] and Holt et al. [180], where citrate concentrations were higher when cows grazed outdoors at pasture.

Feed has a greater influence on the fat soluble vitamins in milk, with the exclusion of vitamin k [174,188]. The concentration of fat soluble vitamins is lower when feed consists of grass silage, maize silage or hay, caused by the lower concentrations of vitamins and carotenoids (vitamin A precursor) in these conserved forages in comparison to fresh grass [188,189,190]. Grass silage was also demonstrated to produce milk with a higher vitamin E content than hay [191]. In terms of water soluble vitamins, group B vitamins are primarily synthesised in the rumen by microbial action [192]. They have however demonstrated correlations with dietary intake. Both vitamin B1 (thiamine), vitamin B2 (riboflavin) and vitamin B7 (biotin) are present in higher proportions in skimmed milk powder (SMP) produced by milk derived from pasture diets (perennial ryegrass and perennial ryegrass with white clover) than a TMR diet [193]. The reduced synthesis of thiamine and biotin in cows consuming TMR is for the most part caused by the more acidic conditions present [194,195]. These acidic conditions are influenced by the higher proportions of concentrates fed in a TMR diet, which are known to reduce ruminal pH [196]. Increasing the forage to concentrate ratio of the animals dietary intake has therefore been shown to increase biotin concentrations in milk [194]. The larger proportion of riboflavin in milk derived from pasture-based diets was attributed to its presence in noticeably larger proportions in grass than in cereal grains [193]. Larger vitamin B3 (in both nicotinic acid and nicotinamide forms) content has been exhibited in TMR derived milks than milks derived from pasture-based diets as a result of cereal grains being a large dietary source of vitamin B3 [193,197].

The correlation of riboflavin and time of year has been observed through its increase during the spring flush from their indoor diet to an outdoor pasture diet until early summer [173]. Vitamins A, D and E have shown similar correlations [190,198], with vitamin D being accredited to the influence of solar radiation, increasing the proportion of vitamin D in milk in summer by up to 4 times that produced in winter [199]. Higher concentrations of vitamin C have been reported in winter in comparison to summer [173]. Calderón et al. [200] identified an increase in vitamin E as cows advanced from early lactation to the beginning of mid lactation, however, vitamin A decreased over the same time period.

Gulati et al. [32] established a positive correlation between SOL and calcium content, particularly in late lactation. The same trend occurred with phosphorus in milks derived from both clover and TMR diets, but it was not statistically evident when perennial ryegrass was consumed. Keogh et al. [185] and Dunshea et al. [201] both identified similar trends for phosphorus. Magnesium and sodium also had larger contents in late lactation [32]. Li et al. [24] similarly acknowledged calcium’s increasing concentration approaching late lactation while Dunshea et al. [201] reported this correlation to occur between 150 and 300 days postpartum. The increasing proportions of the majority of these minerals is due to the tight junctions between mammary epithelial cells becoming less effective as each lactation progresses [169]. Unlike other minerals, citrate is unable to permeate the mammary epithelium in either direction, but is instead produced in milk by exocytosis of Golgi vesicles [202]. The citrate content therefore does not increase as the mammary epithelium loosen but instead decrease from early lactation until late lactation [203]. The proportion of iodine in milk is inversely proportional with milk yield, therefore as the animal approaches peak milk yield in early lactation, iodine is at its lowest concentration before increasing as lactation progresses [204].

Other influences on mineral content include health and genetics. Studies from both Sloth et al. [205] and Garnsworthy et al. [203] suggested udder health had very little impact on the milk citrate content. Mastitis had a much larger effect on most of the other minerals in milk with reductions to most minerals identified apart from sodium and chloride ions whose concentrations both strongly increased [177]. Fox et al. [22] categorized milk from jerseys as possessing larger quantities of calcium and phosphorus than breeds such as Holstein, but also produce lower proportions of sodium and chloride than Holsteins.

## 3. Functionality

### 3.1. Milk pH

The pH of milk is one of the most critical considerations of milk manufacturing, particularly due to its role in the gelation of various dairy products, and its role in detecting milk quality through acid titration testing and heat stability. Slight variations in pH have been hypothesized to impact greatly the physiochemical parameters of the milk constituents. The dissolved Ca content of milk serum for example is reduced when pH increases due to the formation of colloidal calcium compounds [127]. As pH declines, reductions were observed in the net surface charge of the micelle and disassociation of casein [206,207]. This disruption to the casein micelles directly affects the rennet coagulation properties, most notably, the rennet coagulation time. A lower pH can also reduce the rennet coagulation time of milk through an increased enzymatic cleavage of caseins, accelerating the necessary reactions [127,208,209,210]. The pH of bovine milk naturally increases under udder inflammation and increased cell counts due to the various components passing through the weakened mammary epithelial cells into the milk [209,211]. This can vary from season to season with autumn milk considered to have a lower pH than both spring and summer despite no clear seasonal trend in the buffering capacity [206]. Li et al. [24] perceived a higher pH in the milk from the late season of a seasonal herd which was correlated with the higher somatic cell count during this period.

### 3.2. Heat Coagulation Time

The heat coagulation time (HCT) or heat stability of milk can be analysed by adding a sample of milk to a sealed test tube which is then placed in a temperature-controlled oil bath. The purpose of a heat coagulation test is to identify the stability of a milk sample and its resistance to coagulation during heat treatment for sterilization. The avoidance of coagulation/gelation is particularly important in many dairy processes including powder production, UHT (ultra-high temperature) treatment, sterilization and preparation of formulated ingredients such as infant formula. Therefore, highly stable milk is required in these cases [212]. A sample with a short HCT is considered unstable and may result in problems during processing and/or a poor product after heat treatment, as opposed to a longer HCT which has a high stability and is ideal for heat treatment processes used to produce products with a longer shelf life including UHT milks and creams [213]. A sample with a poor HCT occurs as a result of a larger colloidal instability of the casein micelles during heat treatment [214]. Larger proportions of κ-casein (due to smaller casein micelle size) [207], higher calcium content [22,215], lower citrate content [22], lower phosphate content [216], and lower proportions of urea [149,217], all reduce the stability of the colloidal casein micelles, reducing the HCT. First stages of coagulation in bovine milk begin to occur between a pH of 5.5 and 6.0 [218]. As the pH of milk begins to approach this range, the proteins begin to charge, resulting in changes to the casein micelles, as well as to the calcium ion concentration in the serum phase [219].

The dissociation of κ-casein from the micelle during heating renders the micelle more susceptible to calcium induced coagulation [207,220], particularly in smaller micelles whose larger surface area to volume ratio increases the proportion of κ-casein present [207]. When higher proportions of calcium are present, the HCT decreases, as a result of the ions transferring to the colloidal phase as calcium phosphate during heating, which reduces HCT by up to 40% [22,215]. The high level of urea in NPN (~55%) causes a positive association between NPN and HCT [221]. Urea has been identified as the main influence of stability during heating because of its thermal degradation to ammonia at high temperatures, increasing pH buffering and decreasing the reduction effect on pH caused by high temperatures [221,222]. Increasing polyvalent anions like citrates and phosphates positively impacts HCT but is only poorly correlated to longer times [22]. Citrates ability to readily bind to Ca^2+^, improving their stability through the reduction of their activity, improves HCT [186,212]. Additional phosphate causes increases of serum casein through dissociation of casein from the micelle, resulting in an increase in HCT [216].

In a study by Kelly et al. [217], seasonal changes in the animal feeding pattern proved to have a larger influence on HCT than SOL effects as evident in milk produced between November and March (which coincides with the time period for which cows are usually indoors). Milk produced during this period had a shorter HCT when compared to that produced during the remainder of the year (April to October). Holt et al. [149] similarly noted urea concentrations accounted for the majority of variation in HCT throughout the year, again finding the difference in diet between cows at pasture in summer and autumn produced milk with a higher HCT than those that were indoors for winter in south-west Scotland. A more recent study by Lin et al. [221] found that introducing autumn calved cows into the spring calving heard negates the seasonal trends in HCT because of the statistically insignificant variance in total protein, protein composition, total calcium and pH. The higher urea content of pasture-based milk results in a considerably higher heat stability than milk derived from dry feed [22].

### 3.3. Gelation

Gelation of milk is used to create products such as cheese and yoghurt and occurs due to destabilization of milk proteins, which generally is induced through two primary reactions, renneting and/or acidification [223]. Low stability milks are quite beneficial in these processes due to their higher affinity for coagulation and gelation.

Fermented dairy products (e.g., yoghurt, sour cream) are produced through the gradual acidification of casein gels. During formation of acid induced gels, Ca and P begin to dissociate from the micellar proteins and become present in the serum, thus increasing the serum ionic strength but lowering the strength of the gels produced [224,225]. These minerals can prevent casein micelles from aggregating due to their binding to the ‘hairy layer’, causing highly repulsive forces between them [226]. Reductions in the gel strength of acid induced structures have been observed when a larger proportion of smaller native MFG [52] and a larger casein/whey protein ratio are present in the gel [227]. The elasticity of the gels and the aggregates within are also influenced by the casein/whey ratio and the whey/κ-casein ratio [228].

Rennet gelation primarily targets the κ-casein within the casein micelle, destabilizing the micelle through the cleaving of κ-casein into para κ-casein and caseino-macropeptide (CMP) [229]. The diffusion of the hydrophilic CMP into the milk serum reduces the electrostatic repulsion in the micelle, promoting the aggregation of para-casein [227]. The reactions of the resultant casein within the micelles accompanied by the CCP dictate gel formation [230]. Minimum proportions of each were required to promote hydrophobic interactions and salt bridging, through the casein molecules and the CCP respectfully, to encourage the onset of gelation [230]. This essentially gives the balance of electrostatic and hydrophobic reactions required to determine the properties of casein gels [231].

The impact protein has on gel strength is slightly contradictory. Results from Glantz et al. [123] suggest high protein content reduces gel strength but improves gelation time while research from Hallén et al. [232] and Guinee et al. [233] associated it with both a firmer curd and a shorter gelation time. Protein quality may be the important factor when looking to improve milk coagulation properties [233]. This is exemplified through the adjustment of protein constituents with higher casein content proving to reduce gelation time while again producing a firmer curd [234]. Glantz et al. [123] acknowledged the role of the larger surface area associated with smaller casein micelles in the production of firmer curds, while also noticing the enhanced gelation properties associated with higher lactose content. Improving the milk fat content while keeping protein concentration constant improved gel time and increased firmness, particularly with larger fat globules [233,235]. Reducing the ionic strength has also been linked with a positive effect on gelation parameters, increasing firmness while also expelling a larger quantity of water from the gel through syneresis [234].

Feed composition and quality has a substantial impact in varying the relative proportions of milks components, altering the gelling properties associated with it. The substitution of straw for the more nutritionally abundant grain resulted in a much improved syneresis of the coagulum formed resulting in a lower moisture content [23]. Lower moisture contents were also noticed when cows consumed high quality pasture [236]. Rennet coagulation times are directly affected by cows transitioning from indoor winter diets out to grass during the “spring flush”, with cheese production sometimes halted completely due to its inability to form a desirable coagulum [237]. Grandison et al. [238] noticed that the curd firmness of the coagulum increased following the spring flush transition. The curd firmness in this study was correlated to increases in casein and mineral concentrations. Green et al. [239] also observed variations in curd firmness with changes to the casein content of milk. O’Callaghan et al. [240] demonstrated that softer gels were produced by pasture derived milks at room temperatures and although not significant, were also softer at refrigerated temperatures than their indoor TMR based equivalents. Grimley et al. [237] also notes how the effects of climatic conditions such as heat stress and compositional changes to pasture could affect coagulating parameters, demonstrated in a later study by Bernabucci et al. [159] in Italy who found a larger abundance of protein fractions through proteolysis, resulting in poorer coagulation properties.

Countries such as Ireland and New Zealand use milk from spring calving herds exclusively to manufacture dairy products such as cheese and yoghurt [167]. This seasonal milk supply has triggered issues for manufacturers because of the changing milk characteristics incurred through the synchronized SOL of the herds. This is particularly evident in late lactation when milk yield is reduced and the proportion of milk components increase. This has resulted in the production of poorer quality products during late autumn and winter such as high moisture cheddar cheese [23,241]. Despite the high proportions of casein in late lactation, the coinciding low levels of lactose and high levels of serum casein [241] during this period can cause a reduction of milk fat recovered in cheese [242], a higher moisture content of cheese, impaired syneresis of the curd [241], and an less firm curd [243], particularly in high somatic cell count milk. In a study where no significant seasonal variation in the casein to total protein ratio was found, Li et al. [244] identified desirable acid gel properties to include a higher gel strength, a higher gelation pH and a shorter gelation time, the opposite to that derived in late lactation milk in this study. The authors attributed the poorer acid gel properties in late lactation milk to the lower proportions α-lac, as well as higher proportions of total calcium, serum calcium, κ-casein, glycosylated-κ-casein and β-lg present, in comparison to that derived in early and mid-lactation [244]. Guinee et al. [245] discovered no significant difference in rennet coagulation properties in milk from late lactation between 218 days postpartum and 284 days postpartum, attributed to the relatively high lactose and protein contents as well as the low somatic cell count values. This suggests the role of good udder health and a high quality diet in the late season could produce milk of a high enough quality for manufacture. Auldist et al. [243] also acknowledged the influence of low somatic cell count in late lactation and its influence on coagulation. Lin et al. [221] observed the introduction of an autumn calving milk serum to that of a spring calving herd and found it reduced the variation in seasonal rennet gelation characteristics to an insignificant level, suggesting that the milk could be used for cheese or milk powder manufacture on a year round basis. Similarly, in a year round calving heard in Reading, U.K., the samples collected during the year suggested the milk should have no difficulties in terms of processing at any point throughout this period [206].

Other influences on the gelation properties of milk include the genetic variations of κ-casein. Cows producing milk with the κ-casein BB allele produced firmer curds, but this allele is less common in Holsteins [127,232]. The BB allele increased the production of casein but derived less whey than cows with the AA or AB allele in studies from both new Zealand [246] and Australia [247], causing discrepancies in the cheese yield per kilo of milk [248].

### 3.4. Foaming Ability

Approximately 50% of the composition of foam based dairy products, which include; whipped cream, cappuccino foams and frozen desserts, are air bubbles and the absorbed milk components they entail. Optimising the foaming traits of these products is primarily dependent on the composition of the dairy products used, particularly the protein content. Increasing the protein content in the dairy product shows positive correlations towards both the foam overrun and foam stability due to the faster dynamic surface tension decrease [249]. MacRitchie [250] accredits proteins low molecular weight and its mixture of both polar and nonpolar side chains for its ability to be readily adsorbed in fluids, giving it a strongly amphipathic nature and thus is readily available for foaming at the air/water interface. The surface activity of these proteins results in a reduction in tension at fluid interfaces, enhancing foaming ability of the fluid [251]. This, along with both the viscosity of the solution and the difference in surface tension between the solution and solvent are the three primary influences on foaming ability and stability of milk [252].

The considerably hydrophobic characteristics of β-casein results in much greater enrichment to the foam phase in comparison to casein micelles and whey proteins [253]. The dissociation of the CCP hinders the structural role of Ca^2+^, which allows a larger proportion of non-micellar casein available for adsorption [254]. The poorer enrichment of whey proteins is due to the fact their hydrophobic residues are buried deep into the structures in contrast to the caseins who’s lack of secondary and tertiary structures leaves their hydrophobic residues exposed at the surface [22]. These globular whey proteins, in particular β-lactoglobulin, produce more viscoelastic films in comparison to casein, due to the thin lamina of bubbles produced by casein foams [22,131]. The denaturation of these globular proteins into protein polymers allow a much higher intrinsic viscosity, decreases air phase volume and, through a reduction in drainage rate, increases foam stability [131]. A negative correlation was associated with FFA and foam stability but FFA did have a positive effect on the coarseness of foams [77]. These FFA are more surface active than proteins, thus, allowing them to dominate the air-liquid interface and become more readily available for foaming [77,255]. This disrupts the protein-protein bonds and therefore weakens the elastic film encompassing the air bubbles, allowing coalescence to occur [77,255,256,257]. Improvements in the ionic strength of rehydrated milk products resulted in an improved whippability because of the increased fraction of free-migrating caseins [253].

In a UK herd of cows calved year round, milk produced in spring had the greatest ability to foam in comparison to the other three seasons, resulting in a positive correlation with protein and casein content during this period [206]. A larger proportion of citrate in spring milk was also correlated to the much improved foam capacity in this study [206]. Augustin et al. [254] similarly noticed the improvements citrate makes to foam overrun and stability in skim milk powder. A higher proportion of SCC in milk has a negative effect on the foaming capacity of milk due to the increased plasmin concentration caused by more permeable mammary epithelial cells [258]. The foaming characteristics of milk can also be improved through processes such as homogenization, allowing the release of foam-promoting proteins from the MFGM [22]. The adsorption of proteins into the foam can be improved through an increase in the temperature at which milk is foamed, allowing for the formation of an improved foam structure [77].

### 3.5. Casein Micelle Size

Casein micelle size is important to manufacturers as it can influence several processes in milk, particularly rennet induced gelation [125]. Feeding system, pH and protein content along with the genetics of the animal have all shown to be stimuli of micellar size [259]. Chen et al. [206] also demonstrated a positive correlation between micellar size and phosphorus content. A study by Devold et al. [259] demonstrated cows on a diet supplemented with ecologically rolled barley had significantly larger micelle size than a group of cows supplemented with commercial concentrates, signifying the association feeding has with casein micelle size. Results from a study by Auldist et al. [260] contrastingly found that altering the quantity of supplemental feed to grazing cows had no significant effect on the average casein micelle size. In Scotland, milk from the summer months produced casein with smaller micellar size than that from winter [261]. Chen et al. [206] similarly noticed casein micelles were up to 5nm smaller during the summer than the winter in the UK but this proved to be statistically insignificant. Lin et al. [221] also observed seasonal variation in casein micelle size in Ireland, however the smallest micelles in this case were observed in spring, contrasting the results by Holt et al. [261] and Chen et al. [206]. Casein micelle size did not incur seasonal variation over the course of two years in a seasonally calved herd in new Zealand [24]. De Kruif et al. [118] similarly found the size distribution of the micelles to be ‘extremely constant’ over the course of a lactation. The genetic variant κ-casein B has been correlated with smaller micelles than the A alternative [125].

### 3.6. Milk Fat Globule Size

The size of the milk fat globule (MFG) produced in the milk of cows usually ranges from <1 µm up to 10 µm in diameter [47]. Each MFG has a different influence on the characteristics and stability of milk products mainly caused by the variation in size, which influences the proportion of MFGM present. When analysing the composition of the droplets, large proportions of SFA present in milk fat have been linked with larger MFG’s reaching up to 4 µm in diameter [262]. Contrastingly MFG sizes ranging from 0.3–1.0 µm generally possessed large proportions of UFA [262]. Smaller globules provide a larger surface area/volume ratio, increasing the proportion of MFGM present, amassing a superior water binding ability and enzymatic content than larger globules [263]. This consequently has an influence on the rennet gelation, melting temperature and taste along with other physical and chemical properties of milk and milk products [45]. The larger surface area of smaller globules creates a superior moisture content within butter, with the higher percentage of water binding membrane material allowing better spreadability [264]. Larger MFG are suitable for butter production due to their lack of stability [49], while smaller MFG caused higher fat loss during churning due to the increased proportion of membrane material they possess, allowing a greater affinity to, and stability in the milk serum [52]. Cheddar cheese’s desirable flavour, texture and colour were found to be associated with larger fat globules [265]. Conversely, Michalski et al. [53] noted how lower lipolysis activity in the smaller globules lead to the superior moisture content in cheese. The presence of larger MFG resulted in firmer curds with a faster curd firming rate in comparison to smaller MFG, when casein size was kept constant [235]. Michalski et al. [50] demonstrated how gel strengths optimum MFG size was dependent on the gelation required. For rennet gels, smaller MFG resulted in firmer gels whereas acid gel strength underwent a significant increase when large MFG were introduced. Smaller MFG have also been recommended in the use of milk for drinking due to their superior stability in milk serum [49].

A linearly proportional relationship can also be seen between the MFG size and the level of dietary fat in the feed consumed [45]. Substituting corn silage with fresh pasture [9] or haylage [266] caused a reduction in the size of MFG produced, with the most influential inclusion at 30% [9]. Increases in dietary fat also increased MFG size, however, this was accompanied with an improved fat yield [45]. Cows fed pasture have also been reported to have a smaller range of globule sizes than those consuming corn silage as part of an indoor TMR diet [267]. Similarly, cows suffering MFD from diets high in UFA produced a smaller average MFG, with a larger ratio of polar lipid membrane to fat globule demonstrated [268]. The increase of polar lipids in smaller fat globules can similarly be accredited to their existence as part of the membrane, whose ratio increases, as the fat globule size is reduced [52].

Due to the limiting synthesis ability of membrane material in the apical plasma, the size of the MFG has been shown to have a strong positive correlation to the concentration of milk fat produced [264]. This limiting factor is the main cause of seasonal trends in MFG size and is highly influenced by the SOL effects towards milk fat yield. A study from Li et al. [24] has similarly demonstrates how MFG size is negatively correlated with the diurnal fat yield of the cow. The negative energy balance experienced by a cow after calving up until peak lactation (~6–8 weeks into lactation) is then converted into a positive energy balance for the remainder of the lactation cycle, providing a larger quantity of membrane material to be readily available to coat the micro lipid droplets, allowing the secretion of smaller fat globules [52]. This has resulted in the secretion of larger globules in early lactation, a reduction in size in mid lactation and the smallest range of globules derived in late lactation milk in spring calving herds [24].

Milk produced from Jerseys showed a greater affinity for short chain FA than both Holsteins and Brown Swiss, allowing a production of larger fat globules in Jerseys than both other breeds [45,52]. The same Jersey cows provided up to 50 times more fat globules with a diameter above 50µm when compared to Holsteins, while also having a much larger average diameter than Friesians and Brown Swiss [45]. A study on 78 Holsteins consuming the same diet has shown that a discrepancy of MFG size can even occur within breeds where a mean globule size varied between 2.5–5.7 µm [268].

## 4. Environmental Impact

Milk production is estimated to contribute between 2.65–2.7% of global greenhouse gas (GHG) emissions [269,270]. Efforts have been made to reduce this figure, with the proportion of GHG emitted per kilogram of milk decreasing by 11% between 2005 and 2015 through increased production efficiency [271]. During the same period however, milk yield increased by 15%, resulting in an overall net increase in GHG emissions [271]. It is therefore essential to take the necessary steps to ensure GHG produced from dairy farming is minimized, particularly when milk demand is expected to increase by 63% by 2050 (compared to 2007 figures) [272]. The predominant GHG produced in dairy farming are methane (CH_4_), carbon dioxide (CO_2_) and nitrous oxide (N_2_O) [19,20,271]. CH_4_ is produced as a result of anaerobic digestion of feed in the rumen as well as from manure storage, CO_2_ emissions as a result of fertilizer application, fuel combustion and electricity generation, and N_2_O as a result of manure deposition, storage and spreading as well as fertilizer application [19,20,271].

Cameron et al. [12] demonstrated how a reduced reliance on TMR for high yielding dairy cattle could both reduce GHG emissions and maintain farm profitability in the United Kingdom. In temperate regions such as Ireland and New Zealand, average performing [273] and high performing [20] grass based dairy systems have been demonstrated to produce milk with a lower carbon footprint compared to confinement TMR systems. Aguirre-Villegas et al. [274] did however demonstrate that the environmental advantages to grazing in regions that cannot support continuous grazing are slight. O’Neill et al. [19] illustrated how the consumption of high quality pasture produced lower quantities of CH_4_ per kg of fat and protein than indoor TMR based systems, despite the higher yield of fat and protein derived from TMR diets. Hristov et al. [275] discussed the influence of increased proportions of dietary fibre intake has on increasing enteric CH_4_ production, as well as the influence of white clover and other legumes in reducing CH_4_ emissions because of their lower fibre content and increased rate of passage through the rumen. Recent studies have highlighted methods of reducing N_2_O emissions from dairy farming such as increasing soil pH [276], incorporating white clover into pasture [277] and the use of slow release urea [278].

Countries have committed to decreasing GHG emissions from agriculture in line with international agreements. Ireland for example, has established an approach involving a marginal abatement cost curve (MACC) [279]. Within this approach, there is an overall strategy for the Irish agricultural industry to target decreased GHG emissions in line with international agreements. Denmark have also enhanced their approach in mitigating GHG emissions by allocating 2 billion Danish Krone between 2020 and 2029 with the aim to reduce GHG emissions from agriculture as part of their ‘National Energy and Climate Plan’ [280]. Furthermore, the European Union’s (EU) Green Deal including the ‘Farm to Fork’ strategy has resulted in development of approaches to reducing the environmental and climate footprint of EU food systems, including the dairy industry, being conceptualized and implemented in the hopes of becoming the first climate-neutral continent by 2050 including a zero pollution ambition [281].

## 5. Conclusions

Understanding the composition of milk is essential to the dairy industry because of its influence on the nutritional value, processing characteristics and the functional properties of dairy products. Different dynamics across the globe have resulted in the use of different calving patterns and feeding systems depending on the most profitable and efficient form of milk production for the region, which has resulted in significant regional variations in milk composition. In areas where farming has intensified, indoor TMR is the most popular system, providing increased dry matter intake to animals and allowing for production with increased yields. TMR also allows for more consistency in regard to milk content because of the equal spread of calving and uniformity of feed throughout the year. This does however result in higher labour, feed and total operating costs, a higher carbon footprint, and raises additional animal welfare issues. Pasture-based dairy provides a system where the benefits outweigh the negatives, despite the compositional and functional variations that can be observed. The compositional and functional changes caused by synchronized SOL pose challenges for manufacturers as a trade-off for an improved nutritional profile of milk, coupled with the improved consumer perceptions and premium price ‘Grass Fed’ dairy demands on the market. This paper provides academics and industry with the tools to understand these variations to a greater extent and therefore capitalize on the positive aspects of pasture-based dairy systems.
